# Prenatal ultrasound findings of the IVC takeoff appearance: Teaching cases highlighting IVC interruption and double vessel sign

**DOI:** 10.1016/j.radcr.2026.06.008

**Published:** 2026-06-29

**Authors:** Bahman Rasuli

**Affiliations:** Department of Radiology, Jame Jam Imaging Center, Arman International Hospital, Tehran, Iran

**Keywords:** IVC takeoff appearance, Inferior vena cava, Interrupted IVC, Double vessel sign, Prenatal ultrasound, Azygos continuation

## Abstract

Familiarity with the relationship between the aorta and inferior vena cava (IVC) in the fetal upper abdomen and thorax are important for evaluating fetal systemic venous anatomy during prenatal ultrasound. In a routine 18-week fetal scan, the IVC Takeoff Appearance was visualized, supporting normal anatomical alignment of the IVC and aorta. In contrast, a 28-week late anomaly scan demonstrated absence of the IVC Takeoff Appearance in a fetus with dextrocardia, raising suspicion for interrupted IVC with azygos continuation and associated double vessel sign. The IVC Takeoff Appearance can be visualized in sagittal and axial planes using a 5 MHz curvilinear transducer, while color Doppler assists in identification of venous flow patterns. These teaching cases demonstrate how the IVC Takeoff Appearance may serve as a useful educational landmark during prenatal ultrasound evaluation of fetal venous anatomy, supporting targeted assessment and recognition of potential IVC anomalies.

## Introduction

Understanding the spatial relationship between the aorta and inferior vena cava (IVC) in the fetal thorax and upper abdomen is important for accurate prenatal assessment of systemic venous anatomy [1,2]. In normal fetal anatomy, the IVC and aorta run parallel below the level of the renal vessels. Beyond this level, the IVC normally deviates anteriorly from the aorta as it courses toward the right atrium [3,4].

The IVC Takeoff Appearance represents the normal anterior deviation of the IVC from the aorta beyond the level of renal vessels. It can be visualized during routine sagittal and axial fetal ultrasound examination using a 5 MHz curvilinear transducer, while color Doppler assists in identification of venous flow patterns [5,6]. The presence of this Appearance supports normal anatomical alignment of the fetal systemic veins, while its absence may raise suspicion for interrupted IVC with azygos continuation. This observation may serve as a useful educational landmark that can prompt targeted evaluation of fetal venous anomalies.

The aim of this manuscript is to present 2 illustrative teaching cases highlighting the IVC Takeoff Appearance as a practical educational landmark for evaluation of fetal systemic venous anatomy, demonstrating how its absence may correlate with the double vessel sign and interrupted IVC with azygos continuation [[1], [2], [3], [4], [5], [6]].

## Case presentation

### Case 1—normal fetal anatomy

A 25-year-old woman presented for a routine fetal ultrasound examination at 18 weeks gestation. Maternal medical history was unremarkable, with no known risk factors or medication exposure during pregnancy.

Ultrasound examination was performed using a 5 MHz curvilinear transducer with sagittal and axial imaging planes. Color Doppler imaging was used to assess fetal venous flow patterns.

**Fig. 1:**
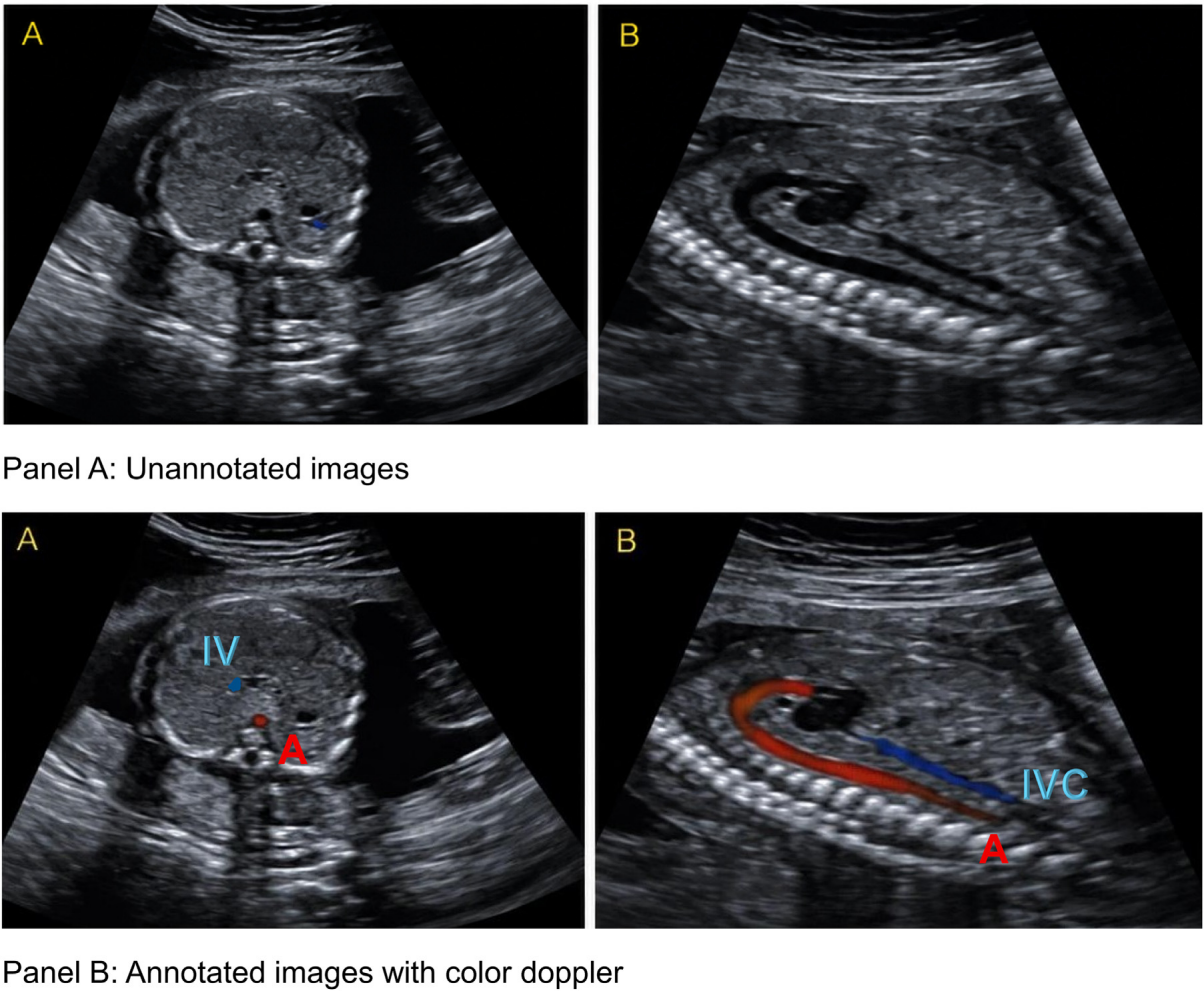
Normal fetal systemic venous anatomy demonstrating the IVC takeoff appearance. Panel A (Unannotated images): axial (A) and sagittal (B) views without annotations. Panel B (annotated images with color Doppler): axial (A) and sagittal (B) views with color Doppler showing the anterior deviation (takeoff) of the inferior vena cava (IVC) from the aorta (AO) after the renal vessels. Abbreviations: AO, aorta; IVC, inferior vena cava.

Imaging demonstrated normal anatomical relationship between the inferior vena cava (IVC) and aorta. In the sagittal plane, the aorta coursed parallel to the spinal column, while the IVC followed a similar course to the level of the renal vessels. Beyond this level, the IVC deviated anteriorly from the aorta, producing the IVC Takeoff Appearance (Fig. 1). Axial views supported this anatomical relationship, while color Doppler demonstrated normal venous within the IVC [7].

This case illustrates the typical appearance of the IVC Takeoff Appearance, supporting normal fetal systemic venous anatomy and serving as a practical educational landmark during prenatal ultrasound evaluation.

### Case 2—IVC interruption with azygos continuation

A 35-year-old primigravida presented for a late fetal anomaly scan at 28-weeks gestation. Maternal history was unremarkable, with no known risk factors or medication exposure during pregnancy. Ultrasound examination was performed using a 5 MHz curvilinear transducer with sagittal and axial imaging planes supplemented by color Doppler evaluation.

**Fig. 2:**
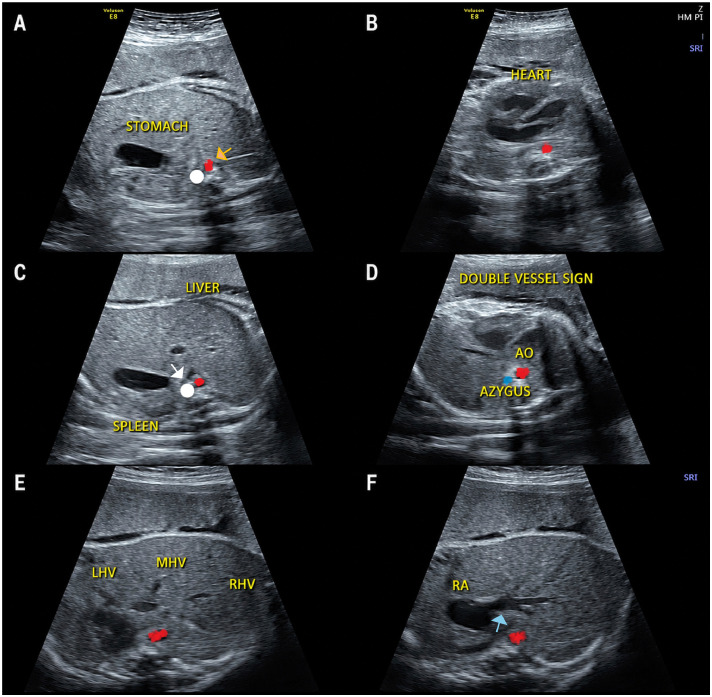
Axial ultrasound images (A and B) show dextrocardia and a right-sided descending aorta (orange arrow). Images (C and D) reveal an absent inferior vena cava (IVC) and a dilated azygos vein (white arrow), indicating azygos continuation of the IVC. Further images (E and F) indicate that the posthepatic segment of the IVC (blue arrow) only drains hepatic veins into the right atrium, with the liver on the right side. All other findings from the fetal anatomical survey are normal. Abbreviations: AO, aorta; IVC, inferior vena cava; LHV, left hepatic vein; MHV, main hepatic vein; RA, right atrium; RHV, right hepatic vein.

Examination demonstrated dextrocardia and a right-sided descending aorta, while the remainder of the fetal anatomical survey was unremarkable. The IVC Takeoff Appearance was absent, and a double vessel sign was identified, with the azygos vein coursing parallel to the aorta (Fig. 2). Color Doppler demonstrated absence of normal flow within the expected IVC region, with compensatory venous drainage through the azygos vein.

Additional observations included:

• The posthepatic IVC segment drained only the hepatic veins into the right atrium.
• No additional major congenital anomalies were identified.
• Fetal situs was otherwise normal, with no sonographic evidence of heterotaxy spectrum disorder.

Postnatal imaging, including echocardiography and abdominal ultrasound, confirmed interrupted intrahepatic IVC with azygos continuation, supporting the prenatal ultrasound findings. The pregnancy resulted in uncomplicated delivery, and the neonate remained clinically stable postnatally.

This case demonstrates how absence of the IVC Takeoff Appearance, combined with the double vessel sign, may serve as a useful educational clue during prenatal ultrasound evaluation, promoting further assessment and counseling regarding fetal IVC anomalies [8,9].

## Discussion

The IVC Takeoff Appearance represents a reproducible sonographic observation that may assist in evaluation of fetal systemic venous anatomy. In normal fetal anatomy, the IVC deviates anteriorly from the aorta beyond the renal vessels, producing the Takeoff Appearance [1,2]. Absence of this appearance may raise suspicion for interrupted IVC with azygos continuation, as illustrated in Case 2 [3,4,6].

Recognition of the IVC Takeoff Appearance together with double vessel sign provides a practical teaching point for sonographers and radiologists during routine prenatal ultrasound evaluation. Identification of these findings may prompt targeted evaluation of the fetal venous system and assessment for associated vascular or cardia anomalies. Although isolated interrupted IVC may be clinically benign, recognition remains important because of potential associations with heterotaxy spectrum disorders, congenital cardiac anomalies and implications for future vascular access or surgical planning [2,3,5].

Visualization of the IVC Takeoff Appearance may be influenced by fetal position, maternal habitus, operator experience, and ultrasound equipment settings. Although a 5 MHz curvilinear transducer is generally sufficient in the mid-to-late second trimester, careful adjustment of imaging planes and use of color Doppler are often necessary to evaluate flow within the IVC and azygos vein. These factors underscore the importance of operator expertise and, when necessary, repeat ultrasound evaluation to support accurate assessment [1,2,4].

Previous studies have described the normal embryologic and sonographic course of the fetal IVC, as well as several variants of interrupted IVC with azygos or hemiazygos continuation [[1], [2], [3], [4], [5], [6]]. Our observations are consistent with prior reports describing the double vessel sign as an important sonographic clue in cases of interrupted IVC with azygos continuation.

Furthermore, distinguishing isolated intrahepatic interruption from combined intrahepatic and suprahepatic interruption is clinically important, as more extensive interruption patterns may demonstrate stronger associations with complex congenital anomalies and heterotaxy spectrum disorders [2,3,5].

These illustrative cases highlight how absence of the Takeoff Appearance may correlate with the double vessel sign, reinforcing its role as an educational sonographic observation rather than a definitive diagnostic marker.

Identification of the IVC Takeoff Appearance during routine prenatal ultrasound may assist evaluation of fetal venous anatomy.

Although the presence of this appearance supports normal IVC anatomy, its absence should prompt careful assessment for venous anomalies, including azygos continuation.

Recognition of these findings may support prenatal counseling and postnatal imaging planning when clinically indicated.

In summary, the IVC Takeoff Appearance and double vessel sign represent practical educational observations during prenatal ultrasound evaluation of fetal venous anatomy. These findings may serve as useful sonographic clues for identifying potential IVC anomalies, supporting targeted imaging evaluation and informed prenatal counseling.

## Conclusion

The IVC Takeoff Appearance is a reproducible sonographic observation that may serve as a practical educational landmark during prenatal ultrasound evaluation of fetal venous anatomy.

Absence of this appearance may raise suspicion for interrupted IVC with azygos continuation, which may be associated with the double vessel sign.

Recognition of these findings may support targeted assessment, prenatal counseling, and planning for postnatal evaluation when clinically appropriate.

Although the Takeoff Appearance is primarily intended as a teaching observation, it may provide a practical and reproducible framework for understanding fetal systemic venous anatomy and prompting further investigation when venous anomalies are suspected.

## Patient consent

The patients provided written informed consents for the publication of this case report, including all clinical data and accompanying images.
